# Sound Classification and Processing of Urban Environments: A Systematic Literature Review

**DOI:** 10.3390/s22228608

**Published:** 2022-11-08

**Authors:** Ana Filipa Rodrigues Nogueira, Hugo S. Oliveira, José J. M. Machado, João Manuel R. S. Tavares

**Affiliations:** 1 Faculdade de Ciências, Universidade do Porto, Rua do Campo Alegre 1021 1055, 4169-007 Porto, Portugal; 2 Faculdade de Engenharia, Universidade do Porto, Rua Dr. Roberto Frias, s/n, 4200-465 Porto, Portugal; 3 Departamento de Engenharia Mecânica, Faculdade de Engenharia, Universidade do Porto, Rua Dr. Roberto Frias, s/n, 4200-465 Porto, Portugal

**Keywords:** audio classification, audio processing, deep learning, Convolutional Neural Networks, transformers, attention mechanisms

## Abstract

Audio recognition can be used in smart cities for security, surveillance, manufacturing, autonomous vehicles, and noise mitigation, just to name a few. However, urban sounds are everyday audio events that occur daily, presenting unstructured characteristics containing different genres of noise and sounds unrelated to the sound event under study, making it a challenging problem. Therefore, the main objective of this literature review is to summarize the most recent works on this subject to understand the current approaches and identify their limitations. Based on the reviewed articles, it can be realized that Deep Learning (DL) architectures, attention mechanisms, data augmentation techniques, and pretraining are the most crucial factors to consider while creating an efficient sound classification model. The best-found results were obtained by Mushtaq and Su, in 2020, using a DenseNet-161 with pretrained weights from ImageNet, and NA-1 and NA-2 as augmentation techniques, which were of 97.98%, 98.52%, and 99.22% for UrbanSound8K, ESC-50, and ESC-10 datasets, respectively. Nonetheless, the use of these models in real-world scenarios has not been properly addressed, so their effectiveness is still questionable in such situations.

## 1. Introduction

As a direct consequence of the growth of the urban population around the world, cities are becoming increasingly more common as human organization structures (Syed et al. [1]). Recently, smart cities are emerging to take advantage of all opportunities that cities can provide to improve the lives of their citizens, such as taking advantage of the sensing architecture spread around the city to create innovative services (Bello et al. [2]). Accordingly, one of the main requirements concerns Urban Sound characterization, which encompasses several tasks, such as sound classification and segmentation, and still poses different challenges (Mushtaq and Su [3], Das et al. [4]). It is estimated that major cities must handle thousands of co-occurring events, with rapid events that require immediate action passing unnoticed by authorities (Mushtaq and Su [3], Das et al. [4]).

Among the environmental sounds, there are several categories, such as natural and human-made sounds, like people speaking and music, and abnormal noises, such as the sounds of gunshots. Urban sounds often associated with human-made noises unrelated to speech or music interpretation are everyday urban noises such as horns and sirens, dog barking and others, which can be classified as abnormal from an urban sound perspective and may require special attention.

Efforts have been made to develop computational algorithms to automatically classify urban sounds acquired at different instants, at the same location or at different ones, extract sound features, and classify a set of particular sounds. However, limitations are still present regarding the combination of multiple classes of sounds, abnormal noise conditions, and a wide range of co-occurring sound events (Mushtaq and Su [3], Das et al. [4,5]).

Earlier sound classification algorithms were traditionally based on handcrafted features (Mu et al. [6], Giannakopoulos et al. [7], Luz et al. [8], Gong et al. [9]). Recently, the proposed algorithms have been based on DL approaches, with the most successful DL architectures being the Convolutional Neural Network (CNN) (Mu et al. [6], Luz et al. [8]) and recently the Transformer (Akbari et al. [10], Elliott et al. [11], Wyatt et al. [12], Park et al. [13], Koutini et al. [14]) architectures. In a CNN, the data are propagated through the included layers via convolutions and other operations, e.g., pooling, flattening, and dropout, having the network the ability to learn both local- and high-level information on the image space (Giannakopoulos et al. [7], Luz et al. [8]). Sound classification based on CNNs has already been proposed, with most of the current approaches exploring the use of pretrained CNNs by redefining the last layers to tackle the sound classification problem (Mushtaq and Su [3], İlker Türker and Aksu [15]) and, recently, using attention models (Akbari et al. [10], Kong et al. [16]) and novel augmentation techniques (Mushtaq and Su [3], Salamon and Bello [17]).

As to urban sound classification, which has as the main objective the detection of relevant sound events acquired from urban scenarios, most of the proposed solutions are based on CNNs. Some works are also based on Recurrent Neural Networks (RNNs) (Kong et al. [16], Gimeno et al. [18]), particularly for sound events that occur in sequence, but should be understood as only one sound event, e.g., footsteps (Kong et al. [16]), by exploring Long-Short Term Memory (LSTM) models (Das et al. [5], Gimeno et al. [18]), and recently attention mechanisms (Zhang et al. [19,20], Qiao et al. [21], Tripathi and Mishra [22], Ristea et al. [23]). However, the optimal architecture for each application has not yet been established, and many opportunities are still available. Furthermore, urban sound understanding has not been addressed properly to operate in real urban scenarios and distributed environments. Therefore, there is a bright future for DL applied to urban sound systems, with huge potential to complement other forms of sensing, such as imaging, which enables multimodal data understanding and applications not yet fully explored (Das et al. [4]).

**Figure 1:**
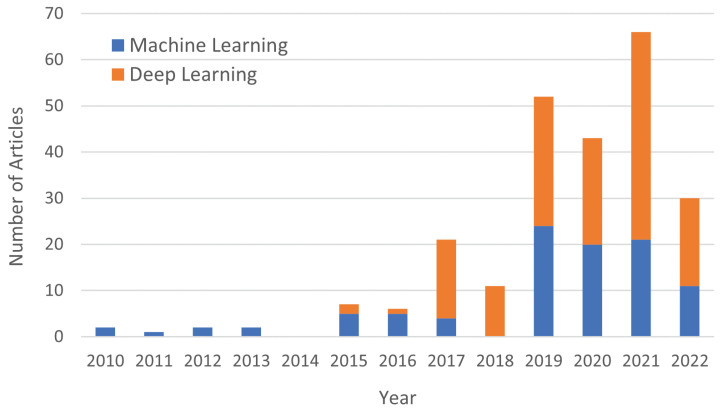
Evolution of the published articles based on deep learning or machine learning approaches found in the Scopus database for environmental and urban sound processing or classification between 2010–2022.

Figure 1 presents the evolution of the number of articles published by year for environmental and urban sound processing or classification. From this figure, it is possible to infer that the growth of DL has gained an increasing interest in the scientific community of this area. Therefore, the most recent proposed approaches are based on DL methods.

Concerning the subject of this study, works aligned in the context of urban sound manifestations are emphasized, namely, works that explore datasets containing urban sound categories such as car horns, sirens, dog bark, and jackhammer, among others. Therefore, this review focuses on classification and audio segmentation methods to characterize urban sound environments. The organization of this article is the following: in Section 2, we describe the methodology employed to search and select the scientific works to be included in this review; in Section 3, we present the findings of this review, mainly as to the architectures used to perform urban sound classification and as to the different methods employed to segment the audios; in Section 4, we list the applications identified in this review; in Section 5, we present details about the most used datasets; and in the final section, we discuss the main conclusions and point out the most promising lines of research.

## 2. Systematic Literature Review—Methodology

This section describes the methodology used to search and select the state-of-the-art works included in this review. The major goal was to gain insight into the significant current research on environmental and urban sound processing and classification. In addition, the following additional features were taken into account:

- Used datasets.
- Proposed models’ architecture, particularly if it is original or modified.
- Metrics used to evaluate the models’ performance.

### 2.1. Search Method

**Figure 2:**
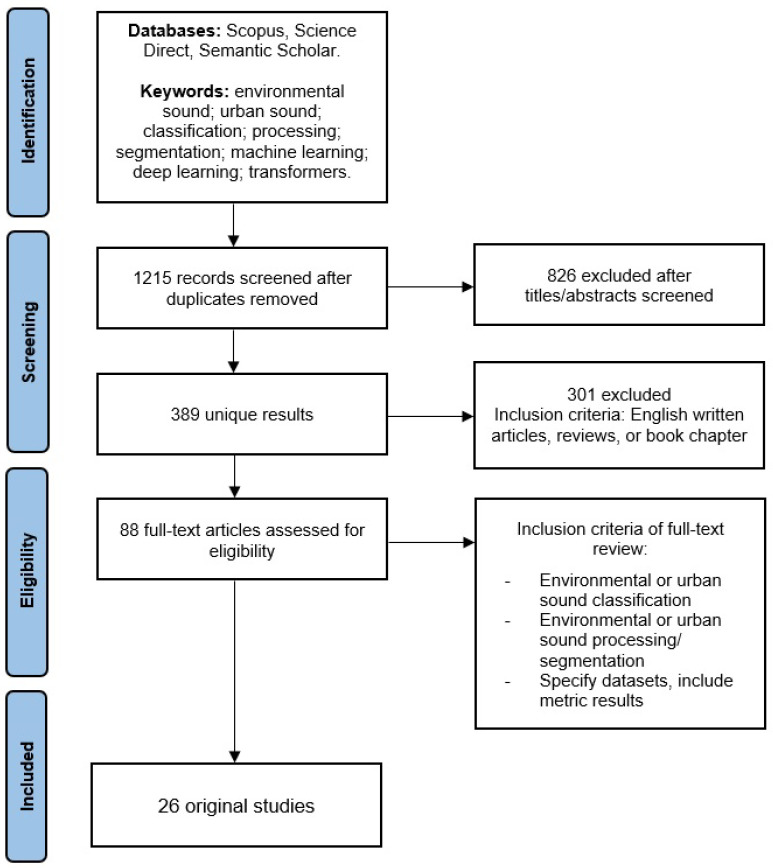
PRISMA diagram of the performed literature search process (adapted from Page et al. [24]).

A systematic literature search was conducted from June 2022 to September 2022 using Scopus, Science Direct and Semantic Scholar databases with distinct combinations of the ensuing keywords: “environmental sound”, “urban sound”, “classification”, “processing”, “segmentation”, “machine learning”, “deep learning”, and “Transformers”. After removing duplicated results, 1215 unique results were produced. An inspection of the title and abstract led to the exclusion of 826 studies for being utterly unrelated to the subject of study. Of the remaining studies, 301 works were excluded by applying the following criteria: studies written in English and peer-reviewed studies, i.e., research articles, literature reviews and book chapters. Finally, by carefully reviewing the body of the text of each remaining work, only 26 studies were maintained. Figure 2 presents the Preferred Reporting Items for Systematic Reviews and Meta-Analyses (PRISMA) diagram of the performed systematic search process.

### 2.2. Sound Classification Methods

Sound classification methods can be applied in several areas, including surveillance, noise mitigation, and context-aware computing, just to name a few. Therefore, to most accurately attribute a class to a specific sound, several Machine Learning (ML) models have been developed to extract the nuclear characteristics of the audio samples under study during training and then classify unseen audios highly confidently.

#### Neural Networks

Researchers have identified some limitations that prevented them from obtaining good results on sound classification tasks. Therefore, Salamon and Bello [17] employed a Deep Convolutional Neural Network (DCNN) in combination with data augmenting techniques, namely noise injection, shifting time, and changing pitch and speed, among the training set to solve the scarcity of labeled data. On the other hand, Das et al. [5] used an LSTM in combination with spectral features obtained from the audio training segments.

Das et al. [4] explored the use of a CNN model with a specific Additive Angular Margin Loss (AAML) and more commonly used stacked features, Mel Frequency Cepstral Coefficients (MFCC) and Chromagram in combination with a CNN. Zinemanas et al. [25] used an Audio Prototype Network (APNet) model composed by an autoencoder and a classifier. Mu et al. [6] introduced a CNN-based model associated with attention mechanisms, called Temporal-frequency attention based Convolutional Neural Network (TFCNN).

## 3. Research Results

**Figure 3:**
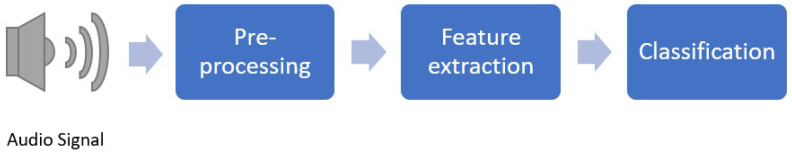
Typical sound classification process.

After revising all the works found on the subject under study, it was possible to understand that there are three fundamental steps to perform sound classification: preprocessing, feature extraction, and classification, as shown in Figure 3. Therefore, this literature review presents the most promising works to address each step.

The objective of the models is to have good generalization performance for unseen data, which commonly requires large quantities of data to train the models effectively. To deal with the scarcity of labeled data for Environmental Sound Classification (ESC), Salamon and Bello [17] proposed four different augmenting techniques to apply to the original training set:

- Time stretching: slows down or speeds up the audio samples, but the pitch remains unchanged.
- Pitch shifting: the audio samples’ pitch is raised or lowered while keeping the duration unchanged.
- Dynamic range compression: compress the dynamic range of the audio using parameterizations from the Dolby E standard and the Icecast online radio streaming server.
- Background noise addition: mix background sounds’ recordings from different scenes with the audio samples.

Furthermore, a detailed analysis of the different techniques is performed to determine the impact of the various data augmentation techniques on the final accuracy, enabling the quantification of the contributions of each of the data transformations employed on the training data, suggesting that a class-conditional augmentation technique during training would be beneficial.

Moreover, it is necessary to understand which features and models can achieve better accuracy. Das et al. [5] presented a comparative study between a CNN and a LSTM model using different combinations of spectral features. First, the audio signal was preprocessed to reduce the amount of redundant information; the Nyquist–Shannon theorem states that the sample rates should be at least twice the value of the frequency of a continuous waveform. However, to reduce the training time, the downsampling was achieved using the librosa library (McFee et al. [26]) default sampling rate of 22,050 Hz. The next step corresponds to the extraction of spectral features such as MFCC, Melspectrogram, Chroma Short-Term Fourier Transformation (STFT), Chroma Constant Q-transform (CQT), Chroma Energy Normalized Statistics (CENS), Spectral Contrast, and Tonnetz. The extracted spectral features combined with data augmentation techniques of pitch shift, time stretch and pitch shift with time stretch, with the final models employed in the classification of the sound events and with a detailed evaluation of the respective accuracy, led to the following conclusions:

- An increase in the number of epochs led to an exponential decrease in the validation error for training and testing data.
- LSTM model had better performance, in most cases than the CNN, which becomes more notable with the data augmentation techniques because the LSTM memory cell encompasses constant error backpropagation, which allows dealing better with noisy data.
- Focusing on the influence of the different used features, the one which led to the best accuracy was the MFCC; however, it was possible to outperform the achieved accuracy by using a stack of different features, mainly of MFCC and Chroma STFT.

Besides the concerns with the type of model and the features that are the best performing ones, it is also necessary to consider the used loss function, which can limit the potential classification accuracy of the used model. Das et al. [4] evaluated different loss functions such as Softmax loss, angular Softmax loss, large margin cosine loss, and additive angular margin loss on the model’s final accuracy. As input, the MFCC features used alone, and the stacked features of MFCC and Chromagram were compared in terms of the model’s final accuracy. A detailed analysis of the results showed a significant improvement in the performance of the used models when the features were stacked together. Besides, it was possible to conclude that there was a boost in the accuracy when a modified Softmax loss function was used in comparison with the Softmax loss function, which led to the best model found: the CNN-based model with additive angular margin loss and the stacked features of MFCC and Chroma STFT as input data.

In addition, it is essential to make the model predictable to identify which input parameter drives the model’s decisions and reduce future malfunctions. To achieve this, Zinemanas et al. [25] proposed an APNet composed essentially of two parts: an autoencoder and a classifier. The autoencoder was composed of the encoder, formed by three convolutional layers. Following the initial two convolutional layers, max-pooling layers were applied to get features at distinct time–frequency resolutions, and the decoder part was formed by three transpose convolutional layers that allowed obtaining audios with great quality in the reconstruction path by minimising the reconstruction error given by the Euclidean mean square loss function about its input and output. Then, the classifier comprised three layers: a prototype layer, a weighted sum layer and a fully connected layer. The prototype layer was responsible for storing a collection of prototypes representing each class and learned in the latent space. To allow the learning of the prototypes in the latent space, it was necessary to minimise the loss function, which happens when there is at least one similar training example for each learned prototype. As a result, training examples agglomerate around prototypes in the latent space. So, this layer outputs a similarity measure based on the separation between each prototype and each instance of encoded data. The similarity measure had two steps: calculating a similarity dependent on frequency and integrating the frequency dimension using a learnable weighted sum. The prototypes can be based on the most important frequency bins, calculated using the square Euclidean distance, followed by a Gaussian function. The frequency-dependent similarity assigns a distinct weight to each frequency bin in the latent space. The frequency dimension is then integrated to obtain *Ŝ*, and by using the following weighted sum:

(1) $$\begin{array}{c} {\hat{S}}_{ij}=\sum_{f=1}^{F}H_{j}\left[f\right]S_{ij}\left[f\right], \end{array}$$

where *H* is the trainable kernel and *F* is the length of the vector for each prototype. The network can learn the optimal way to weigh each frequency bin for each prototype, which is due to the crucial role that the kernel plays in differentiating between overlapping sound classes by concentrating on the most significant frequency bins for each prototype. Finally, the fully connected layer, whose activation function is a Softmax to enable classification, learnt the decisions to convert the similarity measure into the predictions. The bias term was not used because it was expected that the network would give the prototypes associated with the class more weight and produce more interpretable kernel weights. It is also important to refer that the prediction process can be illustrated, even though it is carried out in the latent space, by using Melspectograms of data instances and prototypes which, by applying the decoder function, are converted from the latent space to the time–frequency input representation. Therefore, the network had as input a time–frequency representation of the audio signal, from where the autoencoder transformed into its representation in the latent space of valuable features. Then, the classifier reused the encoded input to make a prediction based on the resemblance between the encoded input and a series of prototype representatives of each class. Accordingly to the previous description, it is possible to understand that this model provides an insight into the decision-making process, allowing the elimination of redundant prototypes and channels, and determining which prototypes are the more representative of each class, enabling to understand which operation is more beneficial for the identification of a specific sound, leading to a direct improvement in the results. However, this model allows interpretation of the decisions, providing a good baseline. Nonetheless, it is not so competitive compared to the noninterpretative models such as CNNs.

As different mechanisms can identify sounds, Mu et al. [6] proposed a TFCNN that, due to the frequency and temporal attention mechanisms, can reduce the impact of background noise and nonrelevant frequency bands. The authors also concluded that the classification performance of transient sounds was enhanced by using temporal attention mechanisms. In contrast, the classification of continuous sounds benefits more from a frequency attention mechanism. Also, the weight combination of both attention mechanisms allowed more attention to the useful information and improved the clarity and distinguishability of the feature distribution of sound events. The model’s architecture consists of attention generation and the backbone network. The generation of attention part seeks that the calculations required for representation learning be concentrated in particular areas. This is accomplished by paying different levels of attention to the frequency band and time frame components of the extracted Log-Melspectrogram from the original data. So, the temporal attention mechanism focuses on the semantically related time frame portion and suppresses noise or silent frames. The frequency attention mechanism assigns more weight to the active frequency bands with distinguishable information. Then, the backbone network part of the model has three layers: a convolutional layer, a pooling layer and a fully connected layer making it possible to extract time–frequency features from the spectrogram processed by the attention mechanism and predict sound phenomenons. The results obtained using this implementation were inferior to the ones of some CNN-based models. Nevertheless, the authors find their model still advantageous since it can ensure high accuracy while requiring little complexity regarding the network’s structure or feature processing.

Table 1 resumes the most relevant aspects of the works found on audio classification using Neural Networks.

### 3.1. Transformers

Other researchers have based their models on attention mechanisms, particularly on a transduction model called Transformer (Vaswani et al. [27]), due to its several advantages such as the total required computational complexity per layer, the quantity of computation that can be parallelized and the path length between long-range dependencies in the network. This section presents some of the most prominent works based on Transformer’s architecture.

Some researchers created models with a hybrid architecture combining Transformers with CNN like Kong et al. [16], who proposed a Convolutional Neural Network Transformer (CNN-Transformer) and an automatic threshold optimization method. Others focused on models based only on Transformers, such as Elliott et al. [11] and Wyatt et al. [12], who presented Bidirectional Encoder Representations from Transformers (BERT) based models capable of performing sound classification at the edge. In the case of Gong et al. [9], an Audio Spectrogram Transformer (AST) was developed, which is a convolutional-free, purely attention-based model able to provide one output for a single channel audio input. Park et al. [13] introduced the Many-to-Many Audio Spectrogram Transformer (M2M-AST), a model based on AST that allows for multichannel audio inputs, multiple output resolution sequences. Akbari et al. [10] presented a Video-Audio-Text Transformer (VATT) and a strategy to decrease the complexity of the training with a slight drop in the final Transformer’s performance: the DropToken technique. Also, to reduce the computational and memory complexity, Koutini et al. [14] introduced a method designated as Patchout.

Motivated by the fact that CNN does not learn the long-time dependencies in a full audio clip, and the labels of audio recordings are typically weak, which requires the right thresholds to detect the sound events under study, Kong et al. [16] developed a CNN-Transformer and an automatic threshold optimization method. The proposed model has a time–frequency representation, Log-Melspectrogram, as input to which a CNN is applied to extract high-level features to get embedding vectors along the time axis. Then, these embedding vectors served as input to the encoder part of the Transformer, allowing the modelling of the dependencies without considering their distance in the input sequence and the parallel computation. Finally, a sigmoid nonlinearity preceded by a fully connected layer is applied to the output of the encoder part of the Transformer to forecast, over time steps, the existence probabilities of the sound classes. To solve the scarcity of strongly labeled data, weakly labeled datasets were proposed to train the model, which can be categorized into two types: segmentwise training and clipwise training. The distinction between them is that the audio clip is divided into numerous segments for segmentwise training, each of which inherits the audio clip’s tags. However, this can result in inaccurate segment tags because the segments may not include the sound event. On the other hand, clipwise training overcomes the preceding issue by learning the tags from a neural network’s hidden layer. Also, it allows the training to be end-to-end with poorly labeled data by aggregating the segmentwise predictions and directly outputting the clip-level prediction. In contrast to segmentwise predictions, latent representations are learned by the neural network. Lastly, thresholds must be applied to the system’s output and optimised to determine whether sound events are present or absent and their onset and offset times. Therefore, the authors have proposed an automatic threshold optimization method to select the optimal thresholds. This method consists of two steps: optimization and evaluation of the system using metrics that do not rely on thresholds, e.g., mean average precision, and next, optimization of the thresholds, for the trained system, regarding a particular metric like F1-score or error rate. The optimization method was tested in several CNN-based models, including the CNN-Transformer, and proved its effectiveness and advantage by improving the results of the models.

However, effective CNN-based models require a vast set of parameters, which is inefficient for working on edge devices, making such models unsuitable for many real-life situations. To carry out the application in real-life cases, Elliott et al. [11] evaluated various audio features extraction techniques on BERT-based Transformers. Then, Wyatt et al. [12] employed a trained BERT-based tiny Transformer on a device with limited resources and deployed it in noisy environments to perform ESC. Both works are based on BERT architecture introduced by Devlin et al. [28], and consist of a multilayer bidirectional Transformer encoder based on the original implementation proposed by Vaswani et al. [27], having as input a given token summed with the position embeddings.

In the work of Elliott et al. [11], the main contribution was the evaluation of Transformers’ performance using several feature extraction methods and their convenience when applied at the edge. Therefore, besides introducing several feature extraction techniques, namely amplitude reshaping, curve tokenization, Vector-quantized varitional autoencoders (VQ-VAE), MFCC, Melspectrogram, and the combination of MFCC, Gammatone Frequency Cepstral Coefficient (GFCC), CQT and Chromagram, the authors also used eleven different augmentation techniques, particularly amplitude clipping, volume amplification, echo, lowpass filter, pitch, partial erase, speed adjust, noise, Harmonic Percussive Source Separation (HPSS), bitwise downsample and sampling rate downsample, on raw audio files. By analysing the obtained results, it was possible to understand that, in general, the various data augmentations techniques lead to better accuracy results and that the best feature extraction method was Melspectrogram, which outperformed all the others with the advantage of being reasonably computationally inexpensive. Regarding MFCC, these models performed slightly better than raw amplitudes, and by adding additional feature extraction methods, the accuracy was improved. However, the cost of computing features using all four feature extraction methods becomes prohibitive, leading to a prolonged training and inference time. The researchers also found that models trained in traditional frameworks have relatively little support for models running in edge devices. The accuracy results obtained with the Transformer based model applied on datasets with a small number of examples per class led to an inferior performance relative to the ones obtained by a CNN-based model.

In the work presented by Wyatt et al. [12], the goal was the development of a robust ESC model capable of working in operational resource-constrained settings. The authors used a Raspberry Pi Zero with poor-quality microphones to record office sounds to fulfil the purpose. The architecture of the used model was based on the design implemented by Elliott et al. [11], which can be divided into three main parts:

- An input transformation baseline allows for choice of the embedding dimension and is constituted of a batch normalization layer, followed by a linear layer. This linear layer allows it to scale up or down one of the dimensions of audio features to a chosen dimension. After passing through the linear layer, a positional embedding is added to the feature vector to incorporate positional information into the prediction.
- A classic Transformer body which is a downsized version of BERT.
- A prediction head, after which three layers: a mean, a linear and a Softmax are used. The mean layer does a global average pooling of the output. The other two layers enable the mapping of the features to output classes before training using cross-entropy loss.

The methodology used to train the model was to employ a dataloader to augment, with an aleatory quantity of noise between a selected noise threshold: a slice of audio randomly selected from each audio file. The amount of noise added varied to make the model robust to high and low signal-to-noise ratios, reducing overfitting. Therefore, the training with noise could generalize to audio without noise, leading to better results than its non-noisy counterpart. The direct incorporation of noise resilience into the model avoided the need to incorporate specific complex noise filters, allowing it to be deployed on thousands of low-power (1W) embedded computational node devices.

**Figure 4:**
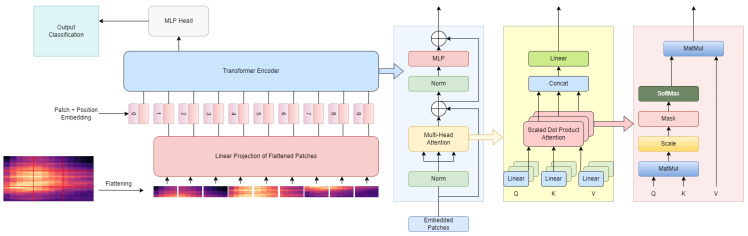
Architecture of Vision Transformer (MLP—Multilayer perceptron).

Subsequently, due to the need to have a Transformer model capable of having competitive results with datasets that have few examples per class, can handle inputs of varying length and do not require architecture changes to perform different tasks, Gong et al. [9] proposed a convolutional-free and entirely based on attention model: the AST model. This model has as input an audio spectrogram and is capable, even in the lowest layers, of capturing long-range global context. Its architecture consists only of the encoder part of the original Transformer’s architecture, which is simple to implement and replicate and makes it easier to perform transfer learning. Furthermore, the formats of audio and images can be addressed in similar ways, allowing cross-modality transfer learning to be used. To accomplish that, it was used an off-the-shelf pretrained Vision Transformer (ViT) (Figure 4), since it has an architecture similar to AST. However, some modifications were needed because ViT input is a 3-channel image. In contrast, AST’s input is a spectrogram with only one channel; therefore, it is necessary for the ViT patch embedding layer’s weights for each of its three input channels to be averaged. Then, they serve as the AST patch embedding layer’s weights. Furthermore, the input audio spectrogram is normalized to ensure that the datasets’ mean and standard deviations are 0 (zero) and 0.5, respectively. Another concern is the positional embedding that, during the training of the ViT, learns to encode the spatial information. Thus, a cut and bilinear interpolate method was proposed to adapt the positional embedding. This method enables the transference of the two-dimensional (2D) spatial knowledge from a pretrained ViT to the AST even if the input shapes are distinct. Lastly, the final classification layer of the ViT is discarded, reinitiating a fresh one for AST. These modifications make it possible for AST to use various pretrained ViT weights for initialization, leading to better results than a randomly initialized AST, which can be more notable when the training bulk is smaller and confirms the reduction in the request for in-domain audio data. The authors also found that Data efficiency image Transformer (DeiT), because it uses data augmentation and a knowledge distillation token, improves data efficiency and model accuracy. Then, regarding the impact of positional embedding adaptation, the importance of transferring spatial knowledge was demonstrated. As for the impact of patch split overlap, it was noticeable that enlarging the overlap length increases the model’s performance and the computational overhead, which grows quadratically. Lastly, regarding patch shape and size, splitting the audio spectrogram into rectangular patches in temporal order achieved better results than splitting it into square patches, which cannot be in a temporal order. However, researchers used squares patches because no pretrained model using the same dataset as ViT and rectangular patches was available. Ultimately, the AST model was tested using various datasets achieving state-of-the-art results while maintaining the same architecture regardless of the input audio length.

Despite producing good results, the previous approach could only produce one audio classification output for single channel input. Thus, to handle a multichannel audio input and have different resolution output sequences, Park et al. [13] proposed the M2M-AST, which is capable of doing sound event localization and detection that comprises the following tasks: direction of arrival estimation and sound event detection. The proposed model has a similar architecture to AST; the only differences are the input feature and the classification token configuration. M2M-AST uses as input features multichannel feature images obtained from 4-channel audio recordings that are segmented into a patch sequence. Then, patch tokens are extracted for each patch using a linear projection. Since the goal was to do sound event localization and detection, the model should output a series of outputs. Thus, patch embedding involved appending a sequence of classification tokens with the same length as the output sequence at the beginning of the patch token sequence. Then, a learnable positional embedding was added to the patch embedding to take advantage of the patch tokens’ position information. The classification token sequence’ outputs compute self-attention between each patch token to learn the audio spectrogram representation. Finally, it uses a dense layer with an activation layer for each of the two tasks. Regarding transfer learning, M2M-AST transfers the weights learned by DeiT; however, some changes were necessary because of the layer learning patch embeddings, which vary in size. Therefore, since DeiT uses 3-channel images as input, for M2M-AST, in the linear projection layer, the weight corresponding to each channel uses the average weight of the three channels in DeiT. Another change was in the positional embeddings for the patch tokens, which are transferred as scaled values via cut and bilinear interpolation to map relative positions of the positional embeddings in DeiT to the input feature. Lastly, some experiments were performed, and the results showed that longer inputs improved both precision and recall, configuring dense patch segmentation with large overlap helped improve performance, and for sound event detection, owing to median filtering, a minor resolution resulted in small performance gains. However, the results did not change considerably for the other task with variations in the output resolution. Finally, soft F-loss performed slightly better than binary cross-entropy for sound event detection. For the direction of arrival estimation, masked Mean Squared Error (MSE) improved performance over binary cross-entropy.

Another concern is to reduce the training time while maintaining competitive results. To address that, Akbari et al. [10] introduced VATT and a technique for reducing training complexity at the expense of a slight decrease in the final Transformers’ performance: the already mentioned DropToken technique. The VATT model is suitable for different downstream tasks in audio, text and video fields. Its architecture is the same as the encoder part of the standard Transformer’s architecture, except for the tokenization and linear projection layer, which is different depending on the modality. Therefore, for each modality, the raw input is projected to an embedding vector in the tokenization layer and fed into a Transformer. However, for the video or audio modality, before feeding the token sequence into the Transformer, it was applied the DropToken technique, where a portion of the tokens is randomly sampled and then only the sampled sequence is given to the Transformer. This technique reduces the computational cost, and the training time, allowing to host of large models on hardware with limited memory. This approach is based on the fact that Transformers’ complexity is strictly related to the input size in a $O(N^{2})$, with DropToken reducing the overall complexity. The model also presents two main settings: the backbone Transformers separated and particular weights for every modality, and the single backbone Transformer applied to any modality with shared weights. In both, the backbone extracts modality-specific representations that are then mapped to common spaces via multilevel projections to allow a comparison with one another using contrastive losses. So, the model optimization is based on the backpropagation of the average loss calculated over a batch of samples. The loss objective used to align video–audio pairs was: Noise Contrastive Estimation (NCE), and to align video–text pairs was: Multiple Instance Learning NCE (MIL-NCE). Regarding the results of several experiments with this model, the researchers concluded that even when the model is shared across modalities, transformers successfully learn semantic video, audio, and text representations. Multimodal self-supervised pretraining has the potential to reduce the reliance on large-scale labeled data. DropToken proved to decrease the pre-training complexity substantially and to have accuracy and training costs comparable to or better than low-resolution inputs for audio and video modalities with little impact on the model’s generalization.

Koutini et al. [14] introduced Patchout, which is a method to reduce the computational and memory complexity for training Transformer models and, in addition, improves the generalization of the models by acting as a regularizer. Therefore, its function is to drop parts of the Transformer’s input sequence during training. First, small overlapping patches are extracted from the input spectrograms to form the Transformer’s input sequence and projected linearly to vectors. Then, the patches are augmented with both frequency and time encoding. Lastly, to reduce the sequence length and regularize the training process, parts of the sequence are randomly dropped during training. However, the whole input sequence is given to the Transformer during inference. Two types of Patchout methods were introduced: the unstructured Patchout, which chooses the patches randomly regardless of their position, and the structured Patchout, which randomly picks some frequency bins or time frames and removes a whole column or row of extracted patches. The researchers also enhanced the models’ performance and prevented overfitting by making use of ImageNet pre-training and some data augmentation techniques such as two-level mix-up, which mixes the final spectrograms with random raw waveforms from the dataset; SpecAugment, which masks up to a certain number of frequency bins and time frames; rolling, which rolls the waveforms randomly over time; and random gain, which multiplies the audio waveform to change the gain by ±7 decibel (DB). Thus, with the development of Patchout, it was possible to train Transformers on audio spectrograms and achieve state-of-the-art results effectively.

Table 2 resumes the most relevant aspects of the works found on audio classification using Transformers.

### 3.2. Sound Segmentation Methods

Proper sound segmentation is a pre-processing step in audio analysis. Its purpose is mainly to identify and properly address the sound categories in a single audio chunk. Usually, the task involves removing unwanted noises or irrelevant sounds to a particular task. To be able to perform audio segmentation, some steps should be followed:

- Feature extraction: The audio input is divided into overlapping frames to allow extraction of the parametric feature vector from each frame.
- Initial detection: It is an optional step where the objective is to remove the silent parts and reject the parts of the signal that are not useful for the task.
- Segmentation: The vector sequence of features is segmented into sub-sequences with common acoustic characteristics. Two main approaches can be employed: distance-based and model-based techniques.
- Post-processing or smoothing: It is also an optional step where the goal is to correct the errors associated with detecting segments with a duration shorter than the specified threshold.

Several metrics and algorithms can be used for the two main techniques mentioned in the segmentation step. Some examples of distance-based metrics are the Euclidean distance, the Bayesian Information Criterion (Neath and Cavanaugh [29]), Kullback Leibler KL2 distance (Joyce [30]), the generalized likelihood ratio (Narasimhan and Mah [31]), and Hotelling’s T2 statistic (Holloway and Dunn [32]). In terms of models, Guassian Mixture Model (GMM), Hidden Markov Model (HMM), Support Vector Machine (SVM), Artificial Neural Network (ANN), Boosting Technology, k-Nearest Neighbor (k-NN), Decision Trees and Fuzzy Logic have been used (Theodorou et al. [33]).

Next, some examples of segmentation based on models are introduced. Tax et al. [34] presented a DCNN model capable of learning the log-scaled Melspectrogram transformation from raw waveform, providing a spectrum visually similar, but slightly smoothed. So, this suggests that upon initializing the first layers of an end-to-end Neural Network classifier with the learned transformation can give comparable results to a model trained on the highly processed Melspectrograms. Besides, due to the capacity of CNNs to approximate complex mappings, it is possible to force the network to learn such transformation implicitly, limiting the need for ad-hoc architectural choices. Therefore, these findings showed that the performance of Neural Network-based models could be improved by incorporating knowledge from established audio signal processing methods.

Martín-Morató et al. [35] discussed two issues caused by the temporal uncertainty of audio events: (1) the generation of errors at the decision level for models trained with precisely annotated strong labels or flawlessly segmented audio events when applied in real-world contexts where weakly segmented audio events and various amounts of background noise exists; and (2) systems trained with weakly labeled datasets deal directly during training with the issue of temporal uncertainty. Therefore, to solve these problems, the authors proposed a pooling layer. This pooling layer employs a non-linear transformation of the learned convolutional feature maps on the temporal axis, which respects a uniform distance sub-sampling criterion in the learned feature space to compensate for irrelevant information of audio events, and enables the information of the actual event to be propagated more effectively through the network. The proposed pooling layer was an advantageous method to learn from weakly labeled data without adding more parameters, and to enhance the robustness in unfavourable scenarios involving significant training and test mismatches.

On the other hand, Gimeno et al. [18] introduced a Bidirectional Long-Short Term Memory (BLSTM) network with a new block incorporated onto the Neural Network, named Combination and Pooling block, which seeks to minimize the repetitive temporal information by using a time pooling mechanism, while learning an appropriate representation through a one-dimensional (1D) convolution layer. Thereafter, the system consists of a Recurrent Neural Network (RNN) based classifier and an HMM re-segmentation module with combinations of Mel log filter bank, chroma features, and also, first and second derivatives, as input. Adding chroma features improves the accuracy of the classification task, as observed in ground truth boundary experiments. On the other hand, the first and second derivatives incorporate the audio signal’s dynamic information, which is more useful for creating the class boundaries than for the classification task. The HMM re-segmentation greatly minimises the system error by enforcing a minimum segment length for the class labels. It also showed that it is advantageous for the segmentation system when the output’s temporal resolution is reduced. The introduction of the Combination and Pooling block allows the implementation of the downsampling within the neural network. Then, to configure the temporal pooling layers, a pooling factor regulates the length of the output sequences in relation to the input length. Consequently, the pooling layers divide an input sequence into several distinct non-overlapping sub-sequences of equal length. After some experiences with multiple configurations changing the layers and the position of the Combination and Pooling block concerning the BLSTM allowed to conclude that having only a pooling layer in between the two BLSTM layers is what makes it possible to achieve better results without adding more parameters, and decreases the computational complexity. Then, to improve the model’s results, it was used a data-agnostic data augmentation routine, the Mixup routine, which creates novel virtual training examples ($\tilde{x}$, $\tilde{y}$) according to:

(2) $$\begin{array}{c} \left\{\begin{array}{c} \tilde{x}=\lambda x_{i}+\left(1-\lambda \right)x_{j},\\ \tilde{y}=\lambda y_{i}+\left(1-\lambda \right)y_{j}, \end{array}\right. \end{array}$$

where ($x_{i}$, $x_{j}$) are two feature vectors extracted at random from the training dataset, ($y_{i}$, $y_{j}$) their respective one hot encoding labels and λ∈[0,1].

Other methods, like the one introduced by Giannakopoulos et al. [7], focus on the feature extraction step. The objective of Giannakopoulos et al. [7] was to employ CNNs as a way of extracting context-aware deep audio features capable of providing additional feature representations to any soundscape analysis classification, which proved, when combined with handcrafted audio features, to give a boost in the classification accuracy without the need for CNN training. The two distinct feature representation steps are combined in an early-fusion scheme and classified using SVM with a Radial Basis Function (RBF) kernel. The handcrafted audio features aim to express the audio signal in a space capable of discriminating an unknown sample concerning the audio classes involved. A set of statistics calculated over short-term audio features is used to represent each signal. Therefore, to process the feature sequence, first, it is necessary to divide the audio signal into mid-term overlapping or non-overlapping windows; then, each one of those is processed by short-term processing, and the feature sequence from each mid-term segment is used for calculating the feature statistics. Each mid-term segment is represented by a group of statistics from the time or frequency domains. Examples of such features used in this work are the zero crossing rate, energy, entropy of energy, spectral centroid, spectral spread, spectral entropy, spectral flux, spectral roll-off, MFCC, chroma vector and chroma deviation. The context-aware deep features were extracted using a supervised CNN that was trained using input spectrograms from the STFT of the segments, allowing them to differentiate between distinct urban context sound classes. Thus, the final fully connected layer’s output was employed as a feature extractor in the initial soundscape classification task. By evaluating the model’s performance on the used datasets, it was possible to show that the combination of handcrafted features with the context-aware deep features culminates in a boost of the model’s results.

Luz et al. [8] proposed a small parameter space CNN model to extract deep features and combine them with handcrafted features. In addition, a feature selection step was used to minimize feature dimensionality, identify redundant and inconsistent features, and recognize which group of handcrafted features can enrich deep features to better discriminate between Urban Sounds. Thus, this step makes the training process faster and less computationally expensive, making it suitable for mobile sound recognition applications or embedded systems. The feature selection experiment outcomes showed that combining perceptual, static, and physical features from frequency and time domains with deep features significantly enhance classification performance.

Table 3 resumes the most relevant aspects of the works found on audio segmentation based on models or/and handcrafted features.

Researchers have shown that deep features include more significant information than handcrafted features, which translates into better results. To further improve the models’ performance, researchers have implemented attention mechanisms that allow focusing on the semantically relevant characteristics. Therefore, the following section is focused on studies that implemented different attention mechanisms.

#### 3.2.1. Attention Mechanisms

Some studies are focused on incorporating attention mechanisms to improve Convolutional Recurrent Neural Networks (CRNN) models’ performance, such as the research works of Zhang et al. [19,20], Qiao et al. [21]. The study presented by Zhang et al. [19] incorporated temporal attention and channel attention mechanisms; later, Zhang et al. [20] used a frame-level attention mechanism. Both proposals used a CRNN model of eight convolution layers to learn high-level representations from the input log-gammatone spectrogram. The channel temporal attention mechanism enhanced the representational power of CNN. Then, two layers of Bidirectional Gated Recurrent Unit (B-GRU) were used to learn the temporal correlation information, to which the CNN-learned features were given as input. Finally, the features are fed into a fully connected layer with Softmax as activation function for the classification task. Also, some data augmentation techniques were used to avoid overfitting, such as time and frequency masking and Mixup techniques. These data augmentation techniques allowed the models to focus on the semantically important frames and produce discriminative features. Also, it allows to reach the following conclusions: to obtain better results, the attention mechanism should be applied to lower layers rather than higher-level layers because the attention mechanism can help preserve the lower-level features that normally carry basic and useful information; applying attention for RNN layers allows to achieve the highest accuracy result; and using sigmoid as scaling function generates better attention weights than Softmax when applying attention at CNN layers. Furthermore, it was possible to understand that temporal attention reduces the impact of background noise. Also, channel attention puts more attention on the filters, which can identify the fundamental characteristics of the sounds. In contrast, frame-level attention focuses on meaningful temporal events while reducing the impact of background noise. Regarding the research of Qiao et al. [21], besides developing a CRNN model with temporal-frequency attention mechanism, the authors proposed a CRNN model using sub-spectrogram segmentation-based feature extraction and score level fusion to highlight the advantages of an attention mechanism. Consequently, the authors showed that the sub-spectrogram segmentation mechanism can consider frequency domain characteristics, but ignores the temporal domain ones. It is important to note that the sub-spectrogram segmentation mechanism truncates the whole spectrogram into a certain number of parts, instead of generating the log Gammatone spectrogram based on the entire frequency band, and using score-level fusion to combine different classification results from different sub-spectrograms. Also, the score level fusion improves the model’s accuracy compared to the uniform weights assignment. Another conclusion is that low-frequency bands contain a big proportion of the characteristics of environmental sounds. However, high-frequency bands contain a few characteristics that are still indispensable for the classification task. On the other hand, concerning the temporal-frequency attention mechanism, the following advantages were highlighted: uses CNN layers to extract temporal-frequency representations from the input log Gammatone spectrogram, shows low complexity despite the capacity to learn more important information from the input, and gives higher accuracy results by focusing on the most critical frames and frequency bands. To conclude, SpecAugmented and Mixup data augmentation techniques were used to enhance the diversity of the training significantly.

Tripathi and Mishra [22] introduced an attention-based Residual Neural Network (ResNet) model that efficiently learns Spatio-temporal relationships in the spectrogram, skipping the irrelevant regions. Regarding the augmentation techniques, the authors used time shift, adding noise and SpecAugment. The proposed attention module allowed the capture of long-range contextual information between the local features of the spectrogram, improved compactness and addressed intra-class inconsistency, which corresponds to the variations between spectrogram features extracted from the different signals belonging to the same class, which can cause performance degradation. In addition, the study revealed that the attention module provided the best accuracy results when affixed after the last residual layer, so higher layers gave more helpful features to define the characteristics of a sound, and the attention module preserves them.

Ristea et al. [23] developed an architecture that employs two Transformer blocks in sequence, the first block attends to tokens within the same frequency bin (vertical axis), and the second one attends to tokens within the same time interval (horizontal axis) of the spectrogram. The proposed approach used noise perturbation, time shifting, speed perturbation, Mixup and SpecAugment as data augmentation techniques. This implementation linearly scales the number of trainable parameters with the input size, which reduces the memory footprint. It can handle high-resolution spectrograms and shown that performing attention only on one axis is insufficient. Thus, combining both attentions gave a considerable performance boost regardless of the chosen order for the vertical and horizontal block. The order only had a marginal influence on the results.

Table 4 resumes the most relevant aspects of the works found on audio processing with attention mechanisms.

The following subsection presents some autoencoder implementations.

#### 3.2.2. Autoencoders

Different types of autoencoders allow a multiplicity of applications, such as the one in the work of Sudo et al. [36], where a multichannel environmental sound segmentation method comprising the following blocks: Feature Extraction, Sound Source Localization and Separation (SSLS), Sound Source Separation and Classification (SSSC) and reconstruction, was implemented. In this work, the feature extraction is performed using the short-time Fourier transform of the initial input signal, the magnitude spectrograms as spectral features, and the sine and cosine interchannel phase difference as spatial features. The SSLS block uses Deeplav3+, which has an encoder-decoder structure and improves the segmentation performance for environmental sounds with different duration. Deeplav3+ allows the extraction of high-level features to predict a spectrogram for every azimuth angle independently of the class. It creates a feature map much smaller than the original spectrogram, allowing it to extract an extensive variety of contexts without adding more parameters. The SSSC block also uses Deeplav3+, but here, the input corresponds to each spectrogram of the output of the SSLS block, inserted one by one. This block surpasses the influence of the spatial features, preventing the network from overfitting the relationship between the direction of arrival and the given class. In conclusion, this approach allows to perform a multichannel environmental sound segmentation without the need to define beforehand the number of sound sources that prevent the overfit between the direction of arrival and the class, which is accomplished by explicitly separating the SSLS and the SSSC block. Also, the SSSC block can separate sound sources coming from a near direction that the SSLS block was unable to separate, which improves the segmentation.

Other approaches do not rely totally on autoencoders, but just on a part of them, for example, only on the encoder part, like the model presented by Venkatesh et al. [37]. The authors proposed a system named You Only Hear Once (YOHO) that predicts the limits of acoustic classes through regression. This model is a CNN, whose architecture is constituted by the MobileNet architecture, which allows the time reduction and frequency dimension by presenting a decoder-like architecture with some extra layers to flatten the last two dimensions. It is also considered a final layer that performs a binary classification that detects the presence, i.e., the start, and the endpoints of an acoustic class segment. The model’s input feature is the Log-Melspectrograms, and the dimension of the input depends not only on the duration of the audio example, but also on the specifications of the Log-Melspectrogram. For the post-processing, to smooth the output and eliminate spurious audio events, threshold-dependent smoothing is used to allow the removal of audio events, whose duration was too short, and the silence segments between consecutive events of the same acoustic class, if they are also too short. In conclusion, this model leads to a fast post-processing and smoothing process due to YOHO’s ability to directly predict the acoustic class boundaries, resulting in a more end-to-end setup. However, it is limited by the time resolution of the input; nevertheless, if the input were raw audio instead of Log-Melspectrogram, the model would be entirely an end-to-end DL approach.

Table 5 resumes the most relevant aspects of the works found on autoencoder-like architecture for environmental sound processing.

Some techniques and steps of audio segmentation had already been referred to in the previous section, such as feature extraction widely used by all the earlier models to predict the class under study. Das et al. [5] used the librosa due to the default sampling rate that allows a reduction in training time. Zinemanas et al. [25] suggested using an autoencoder scheme to allow the extraction of features and predict the class by constructing a latent space more capable of expressing the audio features. Mu et al. [6] introduced self-attention mechanisms, combining a temporal and frequency attention mechanism, to reduce the impact of background noise and non-relevant frequencies and focus on the most important parts of the signal. Besides that, the researchers used Harmonic Percussive Source Separation (HPSS) to separate harmonic and percussive components. For the models based on Transformers, the majority took advantage of feature extraction. However, this step is especially explored by Elliott et al. [11] by suggesting several different techniques, as mentioned in the previous section. Kong et al. [16] presented a post-processing method capable of automatically optimizing the values for the thresholds. Other researchers, such as Akbari et al. [10] and Koutini et al. [14], introduced DropToken and Patchout techniques, respectively, which randomly drop part of the input sequence before feeding it to the Transformer to reduce the training complexity.

Some methods for spectrogram representation envisioning the enhancement of the sound segmentation task are discussed in the following section.

#### 3.2.3. Methods for Spectrogram Representation

In this section, the reviewed works present modifications of the input spectrogram to be supplied to the deep learning models and evaluate several data augmentation techniques. Therefore, Mushtaq and Su [3] presents a logarithmic scale transformation of the Melspectrogram, named L2M, corresponding to the Log(Log-Melspectrogram), and L3M corresponding to the Log(Log(Log-Melspectrogram)). Such parameters are particularly useful for two novel data augmentation techniques: NA-1 and NA-2, based on Spectrogram Image Features (SIF). NA-1 and NA-2 use a single image as a feature at a time, but NA-1 consists of the enhancement of SIF data by combining several audio features based on spectrograms. At the same time, NA-2 is a vertical combination of various accumulated features in the form of spectral images in pairs. Besides, trim silence was used as a pre-processing technique due to the silent parts of the audio clips. Regarding the transfer learning model, Dense Convolutional Network (DenseNet)-161 with ImageNet weights was the chosen classifier, which was further fine-tuned by using individual optimal learning rates combined with discriminative learning.

İlker Türker and Aksu [15] proposed a new time-convexity representation based on graph representations of successive frames after segmentation with constant window and hop-length parameters of the original sound signal. This representation, named Connectogram, is a colourful graph-generator approach that includes three layers, each derived with distinct undersampling rates where the horizontal axis stands for the time, and the vertical axis for the signal fluctuations. So, it is an Red-Green-Blue (RGB) image that the model can utilize as input. This approach seems to carry frequency-related info pairs with amplitude information by having amplitude information of the original sound as vertical fluctuations. The intensity of these fluctuations corresponds to the colours. However, Connectogram is not a competitive representation, but can significantly improve the representation capability of Melspectrograms, if generated with the same segmentation parameters. The best accuracy result was obtained when a combination of two Melspectrogram with different parameters and a Connectogram was used as input for the ResNet50 model. Concerning data augmentation, the input allows having two stages of augmentation, the first regards deformation methods to the raw sounds, and the second includes image distortion methods that are applied to the Connectogram, such as rotation, horizontal and vertical shift, brightness, shear and zoom.

Table 6 resumes the most relevant aspects of the found works that employ new feature extraction techniques.

Table 7 resumes the results of all of the articles included in this literature review.

## 4. Applications

Several areas can benefit from sound classification methods, such as audio surveillance, security, soundscape assessment, and monitoring biomedical, wildlife or urban environments, just to name a few.

Regarding biomedical applications, sound classification methods allow more accurate diagnosis, the detection of abnormal sounds, and for non-invasive and safer monitoring of organ conditions such as lungs (Fraiwan et al. [38]) or heart (Tuncer et al. [39], Er [40], Zeinali and Niaki [41]). For example, Grooby et al. [42] implemented a method to predict the condition of heart and lungs based on a 5-level scale.

Wildlife monitoring is implemented to protect, preserve, and identify animal species and possible problems related to the habitats or animal’s health. For instance, the works of Soares et al. [43], which aimed to detect the queen bee presence to evaluate the hive health, of Shen et al. [44,45], where models to detect pig cough were explored, of Tuncer et al. [46], Zhang and Li [47], Hsu et al. [48] that were focused on birds sounds and of Xie et al. [49,50], Brodie et al. [51] focused on frogs calls with the following purposes: environmental and species monitoring, biodiversity assessment, species identification and animal follow-up. On the other hand, examples of works tackling multiple animal species identification are the ones of Zhong et al. [52], LeBien et al. [53], where species of birds and amphibians were studied, and of Kim et al. [54], where sounds of anurans, birds and insects were distinguished. Moreover, Ghiurcau et al. [55] explored the detection of intruders to preserve wildlife regions. Bedoya et al. [56] and Wang et al. [57] introduced different methods to infer the intensity levels of rainfall in different scenarios. Bedoya et al. [56] used audio recordings in forests, whereas Wang et al. [57] used audios provided by surveillance cameras deployed in urban scenarios.

Urban Sound classification methods are very important for smart cities (Bello et al. [2]), in particular, for audio surveillance (Shreyas et al. [58], Laffitte et al. [59]) and noise pollution mitigation (Arnault et al. [60], Bello et al. [61]). For example, Scarpiniti et al. [62] implemented a method in construction sites to detect hazards and unmanned activity monitoring. Also, robotic scene recognition (Aziz et al. [63]), drones (Ibrahim et al. [64]), siren detection, particularly to allow the priority vehicles to arrive at its destination sooner (Pramanick et al. [65], Fatimah et al. [66]), are some applications that can take advantage of urban sound classification systems.

## 5. Datasets

Among the sound universe exists a wide range of possible sound categories. These sounds can be categorized as urban sounds, speech expression emotions, spoken words, human and animal sounds, and medical sounds, among many others.

Several workshops and challenges regarding sound themes have been especially useful for the research community to develop and test their solutions. One of the widely known workshops is the DCASE [67], yearly organized to address several sound-related thematics categorized into different tasks. These tasks often encompass computational environmental audio analysis, such as acoustic scene classification, sound event detection and localization, audio tagging, and rare audio events detection.

Concerning the subject of this review, the datasets aligned in the context of urban sound manifestations are emphasized, containing abnormal urban sounds such as car horns, dog barking, and drilling. The main characteristics and brief descriptions of the primarily identified datasets are summarized in Table 8.

## 6. Conclusions

Urban sound classification presents unstructured characteristics filled with noise and sounds unrelated to the sound event under study (Mu et al. [6]). Such facts make it a very challenging problem since indoor and outdoor activities are involved. The need to identify confusing acoustic scenes from everyday life, the sometimes multiplicity of overlapping sound events and the existence of numerous sound sources also represent challenges. In addition, the big distance between the sound acquisition’s microphone(s) and the audio source makes the signal-to-noise ratio very small (Mushtaq and Su [3]), which might be considered a drawback.

Usually, sound classification has three main steps: the pre-processing of the input audio signal, the acoustic feature extraction from the pre-processed audio signal and the audio signal classification (Das et al. [5]). Therefore, the researchers have proposed several models to address this challenge, focusing on different steps of the task. Some try to develop or improve the model’s architecture by using modified versions of the loss functions, methods to drop parts of the input sequence, or by exploring various types of architectures such as DCNN, CRNN, LSTM, ResNet, DenseNet and more recently, Transformers. Most of the proposed approaches were based on DL methods because even if it is more challenging to identify which parameters drive the model’s decision, non-interpretative DL models show superior results (Salamon and Bello [17], Zinemanas et al. [25]). Others have concentrated more on the sound segmentation part of the task with approaches ranging from implementing different feature extraction techniques. However, the combination of handcrafted features allows typically to achieve better results (Das et al. [4,5]), particularly when handcrafted features are combined with deep features (Giannakopoulos et al. [7], Luz et al. [8]). Also, the use of model-based techniques to segment the sound, like CNNs or autoencoders, are capable of learning transformations from the raw waveforms and able to provide comparable results to models trained on highly processed features (Tax et al. [34]). Other examples are the introduction of new blocks or layers that reduce redundant information (Gimeno et al. [18], Martín-Morató et al. [35]) or the employment of different attention mechanisms to focus on the semantically relevant characteristics. The ones that combine time and frequency attention mechanisms show more significant improvements.

Furthermore, a problem that researchers discuss is the scarcity of data, and several audio data augmentation techniques are used to solve this problem and avoid overfitting. On the other hand, some researchers have used cross-modality transfer learning by introducing pre-trained models in the image domain. Such an approach allowed them to use the weights knowledge to facilitate and accelerate the training process and enable the implementation of data augmentation techniques from the vision domain.

Regarding the applications, even though many recognize the importance of urban sound classification to improve noise monitoring, surveillance and security systems to provide accurate event detection invaluable time, very few have implemented their methods in real-world applications.

The main objective of this literature review was to summarize the most recent works on the subject to understand the current approaches in this area and identify their problems or limitations. Out of the articles included in this review, the approach proposed by Mushtaq and Su [3] was the one that gave the best results for the most popular ESC datasets: 97.98% for UrbanSound8k, 98.52% for ESC-50 and 99.22% for ESC-10, which were evaluated according to the official splits by doing the k-fold cross-validation evaluation of the results.

This literature review sheds light on the bright future that DL approaches can have, mainly the substantial improvements in the model’s accuracy based on Transformers architecture, indicating that future developments for urban sound characterization will be most likely leveraged by attention mechanisms present in Transformers. Besides, transfer learning showed promising results in all architectures in each it was used. Also, the employment of the models in real-world applications is suggested to assess their feasibility in real-world scenarios better.

## Figures and Tables

**Table 1:** Resume of the research articles found on audio classification using Neural Networks.

Authors/Year	Model Features	Contributions/Benefits	Limitation(s)	Dataset/Metrics
Salamon and Bello (2017) [17]	DCNN combined with data augmentation techniques of time stretching, pitch shifting, dynamic range compression, and background noise.	Overcomes the problem of data scarcity; Shows that DL models produce better results due to their representational power and capacity combined with data augmentation.	Some augmentation techniques have a negative impact on the accuracy of some classes.	UrbanSound8K; Accuracy: 73% without data augmentation and 79% with data augmentation.
Das et al. (2020) [5]	CNN and LSTM models are used with a stack of multiple features as input and data augmentation techniques: pitch shift, time stretch and pitch shift along with time stretch.	Increasing the number of epochs leads to a decrease in the validation error until reaching convergence; LSTM deals better with data noise; The single feature input that allows the best result is MFCC; however, the stack of features of MFCC and Chroma STFT gave the best results out of all input features.	Needs large datasets; Execution time of 37.14 min with a GeForce RTX200 GPU with 6 Gigabytes of VRAM and boost clock of 1.68 GHz, consumes around 8 Gigabytes of RAM to train both models and requires large resources for inference.	UrbanSound8K; Accuracy: 98.81% for LSTM model using data augmentation and a stack of MFCC and Chroma STFT as input.
Das et al. (2021) [27]	CNN model is used with a single feature input (MFCC) and with a stack of features using as loss function a modified Softmax loss function.	The stack of MFCC and Chroma STFT as input provided the best results; A modified Softmax loss function showed to be more beneficial than the Softmax loss function; Additive angular margin loss was the loss function that gave the best results.	The sophisticated loss functions created an intelligible space to separate the different classes due to an increase in the compactness within classes.	UrbanSound8K; Accuracy: 99.60% of CNN model with an additive angular margin loss function without data augmentation.
Zinemanas et al. (2021) [25]	APNet is used with a time–frequency representation of the audio signal as input; prediction based on the resemblance between the encoded input and a collection of prototypes.	Provides insights into the decision-making process, helping the design of better models; Models are more explicit, allowing the possibility to understand what are the prototypes more representative of each class and which operation is more beneficial for identifying a specific sound.	The obtained results are not competitive with a noninterpretative DL model.	Medley-Solos-DB, Google Speech Commands, UrbanSound8K; Accuracy: 65.8% for Medley-Solos-DB, 89% for Google Speech Commands and 76.2% for UrbanSound8K.
Mu et al. (2021) [6]	TFCNN model is used, which is a CNN model with temporal and frequency attention mechanisms.	Attention mechanisms reduced the background noise and nonrelevant frequency bands influence; Low network structure complexity, and plain feature processing.	Did not show a similar improvement for all classes, negatively impacting the correct classification of some classes.	UrbanSound8K, ESC-50; Accuracy: 84.4% for ESC-50 and 93.1% for UrbanSound8K.

**Table 2:** Resume of the research articles found on audio classification using Transformers.

Authors/Year	Model Features	Contributions/Benefits	Limitation(s)	Dataset/Metrics
Kong et al. (2020) [16]	CNN-Transformer model and an automatic threshold optimization method are used.	Computations are done in parallel; Use weakly labeled datasets to train the model and outputs directly clip-level predictions; Automatic threshold optimization is employed.	CNN-based models need many parameters.	DCASE2017 Task4; F1-score: 64.6% for AT, 57.3% for SED; Precision: 69.1% for AT; Recall: 60.7% for AT; Error rate: 68% for SED.
Elliott et al. (2021) [11]	BERT-based Transformer for ESC at the edge is used.	Evaluation of Transformers’ performance using several feature extraction techniques and data augmentation; Enables ESC on edge devices.	Models trained in traditional frameworks have little support to be converted to models that run at the edge; Lower competitive results when trained with small datasets.	ESC-50, Office Sounds; Accuracy: 67.71% for ESC-50, 95.31% for Office Sounds.
Wyatt et al. (2021) [12]	BERT-based Transformer used for ESC on a resource-constrained device applied in noisy environments.	The model trained with noise-augmented data can generalize to audio without noise and prevents having to construct custom acoustic filters to apply the model in real-life environments.	Needs large datasets; Only employed on small edge-end devices.	Office Sounds; Accuracy: 75.4% for non-noisy dataset, 81.2% for noisy dataset, Precision: 76.5% for non-noisy dataset, 79.7% for noisy dataset, Recall: 75.6% for non-noisy dataset, 80.6% for noisy dataset, F1-score: 75% for non-noisy dataset, 80% for noisy dataset.
Gong et al. (2021) [9]	Audio Spectogram Transformer, a purely attention-based audio classification model, is used.	Even in the lowest layers, it can capture long-range global context; Able to handle different input audio lengths without changing the architecture; Few parameters and fast convergence.	Cannot use rectangular patches due to the inexistence of a pre-trained model that used the same dataset as ViT; Unable to use only an AudioSet pre-trained model.	AudioSet, ESC-50, Speech Commands V2; mAP: 48.5% for AudioSet, Accuracy: 95.6% for ESC-50, 98.11% for Speech Commands V2.
Park et al. (2021) [13]	Audio Spectrogram Transformer that can handle various output resolutions is used.	Shows that Soft F-loss performs better than binary cross-entropy; Designed to deal with a multiplicity of output resolutions.	Large model size; Evaluates sound event localization and detection using only one dataset.	TAU-NIGENS Spatial Sound Events 2021; Error rate: 50%, F1-score: 65.7%, Recall dominant score: 74.7%.
Akbari et al. (2021) [10]	Transformers for multimodal self-supervised learning from raw video, audio and text are used.	Learns effectively semantic video, audio and text representations; DropToken technique reduces the pre-training complexity, which reduces computational costs, the training time and enables the hosting of large models on restricted hardware.	Needs large datasets to be trained due to the large size of the network.	Only 2 out of 10 datasets were from the audio domain: ESC-50, AudioSet. mAP: 39.4% for AudioSet, AUC: 97.1% for AudioSet, d-prime: 2.895 for AudioSet, Accuracy: 84.9% for ESC-50.
Koutini et al. (2021) [14]	Audio Transformer with Patchout which optimizes and regularizes Transformers on audio spectrograms is used.	Patchout improves the generalization and reduces the computation and memory complexity.	Increases the training time	AudioSet, OpenMIC, ESC-50, DCASE20; mAP: 49.6% for AudioSet, 84.3% for OpenMIC, Accuracy: 96.8% for ESC-50, 76.3% for DCASE20.

AT—audio tagging, SED—sound event detection, mAP—mean average precision, AUC—area under the receiver operating characteristic curve.

**Table 3:** Resume of the research articles found on audio processing with segmentation based on models or/and handcrafted features.

Authors/Year	Model Features	Contributions/Benefits	Limitation(s)	Dataset/Metrics
Tax et al. (2017) [34]	End-to-end CNN model classifier with the first layers initialized is used.	Training the first layers of a DCNN model using unlabeled data allows it to learn high-level audio representation; Incorporating knowledge from audio processing methods can enhance the performance of Neural Network-based models.	Not able to outperform the models trained on processed features.	ESC-50; Accuracy: around 50%.
Martín-Morató et al. (2020) [35]	CNN-based models with an adaptive pooling layer based on a non-linear transformation of the learned convolutional feature maps on the temporal axis are used.	Distance-based pooling layer to improve CNN-based models for audio classification in adverse scenarios; Allows the systems to a better generalization for mismatching test conditions; Learn more robustly from weakly labeled data; Enables a better propagation of the information about the actual event across the network.	Only uses isolated events with a clear beginning and end.	UrbanSound8K, ESC-30, DCASE2017 T4; Macro-averaging accuracy: 77% for ESC-30, 73.96% for UrbanSound8K, F1-score: 48.3% for DCASE2017 T4, Precision: 68.2% for DCASE2017 T4, Recall: 46.7% for DCASE2017 T4.
Gimeno et al. (2020) [18]	BLSTM with a Combination and Pooling block is used.	A combination of BLSTM modelling capabilities with HMM backend smooths the results and significantly reduces system error; Combination and Pooling block reduces redundant temporal information.	Needs large datasets; The proposed block could not outperform the model with HMM re-segmentation.	3/24 TV, CARTV; Segmentation error rate: 11.80% for 3/24 TV, 24.93% for CARTV, Average class error: 19.25% for 3/24 TV, Accuracy: 16.05% for 3/24 TV.
Giannakopoulos et al. (2019) [7]	CNN is used to extract context-aware deep audio features and combine them in an early-fusion scheme with handcrafted audio features.	Using CNN as a feature extractor can improve the performance of the audio classifier by transference audio contextual knowledge without the need for CNN training.	Low accuracy results.	TUT Acoustic Scene (used to train), UrbanSound8K, ESC-50; Accuracy: 52.2% for ESC-50, 73.1% for UrbanSound8K.
Luz et al. (2021) [8]	CNN model used to extract deep features that are combined with handcrafted features. As classifiers, Support Vector Machine and Random Forest were used.	Feature selection steps to reduce feature dimensionality and understand which handcrafted features could enrich deep features to better distinguish between Urban Sounds; Deep features hold more important information than handcrafted features.	Data augmentation techniques were not evaluated; To extract features from the Melspectrogram, only one not-too-deep CNN model was used.	ESC-10, UrbanSound8K; Accuracy: 86.2% for ESC-10, 96.8% for UrbanSound8K.

**Table 4:** Resume of the research articles found on audio segmentation with attention mechanisms.

Authors/Year	Model Features	Contributions/Benefits	Limitation(s)	Dataset/Metrics
Zhang et al. (2019) [19]	CRNN model with temporal and channel attention mechanisms is used.	The two attention mechanisms enhance CNN’s representation capabilities and lead it to concentrate on the semantically significant portions of the sounds; The attention mechanism allows better outcomes when applied to lower layers rather than the higher-level layers.	Does not quantify the robustness to noise.	ESC-10, ESC-50, DCASE2016; Accuracy: 94.2% for ESC-10, 86.5% for ESC-50, 88.9% for DCASE2016.
Zhang et al. (2020) [20]	Frame-level attention mechanism based on CRNN is used.	The attention model automatically focuses on the semantically relevant frames and produces discriminative features; Low computational complexity.	Does not quantify the robustness to noise.	ESC-50, ESC-10; Accuracy: 93.7% for ESC-10, 86.1% for ESC-50.
Qiao et al. (2021) [21]	CRNN model is used with sub-spectrogram segmentation based feature extraction and score level fusion; CRNN model using temporal-frequency attention.	Score level fusion improves the accuracy in comparison with the uniform weights assignment; Low complexity when generating the temporal-frequency attention map when using the attention mechanism; High accuracy results when using temporal-frequency mechanisms.	Sub-spectrogram segmentation mechanism just considers frequency domain characteristics; Multi-dimensional search spaces are needed to optimize segmentation limits and the number of segments, which are, in general, computationally prohibitive.	ESC-50; Accuracy: 82.1% for ESC-50 with sub-spectrogram segmentation, 86.4% for ESC-50 with temporal-frequency attention.
Tripathi and Mishra (2021) [22]	Attention-guided residual network that efficiently learns spatio-temporal relationships of a signal’s spectrogram is used.	The attention module resolves the intra-class inconsistency; Identifies more semantically relevant parts of the spectrogram and correctly highlights them while providing a visual description.	Does not quantify the robustness to noise.	ESC-10, DCASE 2019 Task-1(A); For augmented datasets: Accuracy: 92.16% for ESC-10, 82.21% for DCASE 2019. For non-augmented datasets: 92% for ESC-10, 82% for DCASE 2019, Precision: 88.70% for ESC-10, 83.47% for DCASE 2019, Recall: 89.80% for ESC-10, 82.28% for DCASE 2019, F1-score: 87.93% for ESC-10, 82.39% for DCASE 2019.
Ristea et al. (2022) [23]	Separable Transformer, which separates the attention for the horizontal axis (time) from the vertical axis (frequency) of spectrograms, is used.	Reduces the number of learnable parameters, which reduces the memory footprint; Able to handle high-resolution spectrograms.	Does not quantify the robustness to noise.	ESC-50, Speech Commands V2, CREMA-D; Accuracy: 70.47% for CREMA-D, 98.51% for Speech Commands V2, 91.13% for ESC-50.

**Table 5:** Resume of the research articles found on audio segmentation with autoencoder-like architecture.

Authors/Year	Model Features	Contributions/Benefits	Limitation(s)	Dataset/Metrics
Sudo (2021) [36]	Multichannel environmental sound segmentation method constituted by a sound source localization block and a sound source separation and classification block is used.	No need to define in advance the number of sound sources; No overfitting between the direction of arrival and the class relationship; Sine and cosine of interchannel phase difference are optimum for sound source localization and separation.	Lack of sufficiently large datasets with separated sound source signals and direction of arrival labels.	The dataset is a combination of 10 datasets resulting in a dataset with 75 classes; Root Mean Square Error: 18.59.
Venkatesh et al. (2021) [37]	YOHO model, which is an end-to-end model with a CNN architecture adapted from the MobileNet architecture, is used.	Converts the detection of acoustic boundaries into a regression problem; The fast inference makes YOHO appropriate for real-time applications; Directly outputs the time boundaries.	Limited by the time resolution of the input.	BBC Radio Devon and MuSpeak, MIREX music-speech detection, TUT Sound Event Detection, Urban-SED; F1-score: 97.22% for BBC Radio Devon and MuSpeak, 90.20% for MIREX, 44% for TUT Sound Event Detection, ≈60% for Urban-SED; Error rate: 75.17% for TUT Sound Event Detection.

**Table 6:** Resume of the research articles found on audio segmentation that introduce new feature extraction techniques.

Authors/Year	Model Features	Contributions/Benefits	Limitation(s)	Dataset/Metrics
Mushtaq and Su (2020) [3]	DenseNet-161 fine-tuned with optimal learning rates and discriminative learning is used.	Introduction of L2M and L3M features; Novel data augmentation techniques: NA-1 and NA-2; Can achieve high results with few training epochs and less quantity of original data.	L2M and L3M are outperformed by other Mel filter-based features. Computationally heavy.	ESC-10, ESC-50, UrbanSound8K (US8K); Accuracy: 99.22% for ESC-10, 98.52% for ESC-50, 97.98% for US8K, Error rate: 0.777% for ESC-10, 1.476% for ESC-50, 2.018% for US8K, F1-score: 99.25% for ESC-10, 98.53% for ESC-50, 98.13% for US8K, Recall: 99.25% for ESC-10, 98.53% for ESC-50, 98.13% for US8K, Precision: 99.24% for ESC-10, 98.57% for ESC-50, 98.14% for US8K, Kappa score, Matthews Correlation Coefficient, False Discovery rate, Fowlkes-Mallows index, Miss rate.
İlker Türker and Aksu (2022) [15]	ResNet50 is used with a combination of two Melspectrogram with different parameters and Connectogram as input.	Introduces a time-convexity graph-based representation for sounds, Connectogram, capable of being fused with Melspectrograms to improve their representation capabilities.	Connectrogram is not a powerful representation when used solely.	ESC-10; Accuracy: 96.46% for ESC-10.

**Table 7:** Results summary of all models included in this literature review.

Authors	Dataset	Accuracy	Other Metrics
Salamon and Bello [17]	UrbanSound8k	79%	-
Das et al. [5]	UrbanSound8k (unofficial splits)	98.81%	-
Das et al. [4]	UrbanSound8k (unofficial splits)	99.60%	-
Zinemanas et al. [25]	UrbanSound8k Google Speech Commands Medley-Solos-DB	76.2% 89% 65.8%	-
Mu et al. [6]	UrbanSound8k ESC-50	93.1% 84.4%	-
Kong et al. [16]	DCASE2017 Task 4	-	AT - F1-score: 64.6%, Precision: 69.1%, Recall: 60.7% Sound Event Detection - F1-score: 57.3%, Error rate: 68%
Elliott et al. [11]	ESC-50 Office Sounds	67.71% 95.31%	-
Wyatt et al. [12]	Office Sounds	81.2%	Precision: 79.7%, Recall: 80.6%, F1-score: 80%
Gong et al. [9]	ESC-50 Speech Commands V2 AudioSet	95.6% 98.11% -	- - mAP: 48.5%
Park et al. [13]	TAU-NIGENS Spatial Sound Events 2021	-	F1-score: 65.7%, Recall: 74.7%, Error rate: 50%
Akbari et al. [10]	ESC-50 AudioSet	84.9% -	- mAP: 39.4%, AUC: 97.1%, d-prime: 2.895
Koutini et al. [14]	ESC-50 AudioSet OpenMIC DCASE20	96.8% - - 76.3%	- mAP: 49.6% mAP: 84.3% -
Tax et al. [34]	ESC-50	≈50%	-
Martín-Morató et al. [35]	UrbanSound8K ESC-30 DCASE2017 T4	73.96% 77% -	- - F1-score: 48.3%, Precision: 68.2%, Recall: 46.7%
Gimeno et al. [18]	3/24 TV CARTV	16.05% -	Segmentation error: 11.80%, Average class error: 19.25% Segmentation error: 24.93%
Giannakopoulos et al. [7]	UrbanSound8K ESC-50	73.1% 52.2%	-
Luz et al. [8]	UrbanSound8K ESC-10	96.8% 86.2%	-
Zhang et al. [19]	ESC-50 ESC-10 DCASE2016	86.5% 94.2% 88.9%	-
Zhang et al. [20]	ESC-50 ESC-10	86.1% 93.7%	-
Qiao et al. [21]	ESC-50	86.4%	-
Tripathi and Mishra [22]	ESC-10 DCASE 2019 Task-1(A)	92.16% 82.21%	-
Ristea et al. [23]	ESC-50 Speech Commands V2 CREMA-D	91.13% 98.51% 70.47%	-
Sudo et al. [36]	75-classes dataset combining 10 datasets	-	Root Mean Square Error: 18.59
Venkatesh et al. [37]	Urban-SED TUT Sound Event Detection BBC Radio Devon and MuSpeak MIREX	-	F1-score: ≈60% F1-score: 44%, Error rate: 75.17% F1-score: 97.22% F1-score: 90.20%
	UrbanSound8K	97.98%	Error rate: 2.018%, F1-score: 98.13%, Recall: 98.13%, Precision: 98.14%, Kappa score: 97.09%, MCC: 97.73%, FDR: 1.854%, FM: 98.14%, Miss rate: 1.863%
Mushtaq and Su [3]	ESC-50	98.52%	Error rate: 1.476%, F1-score: 98.53%, Recall: 98.53%, Precision: 98.57%, Kappa score: 98.95%, MCC: 98.49%, FDR: 1.469%, FM: 98.55%, Miss rate: 1.469%
	ESC-10	99.22%	Error rate: 0.777%, F1-score: 99.25%, Recall: 99.25%, Precision: 99.24%, Kappa score: 98.93%, MCC: 99.13%, FDR: 0.758%, FM: 99.24%, Miss rate: 0.744%
İlker Türker and Aksu [15]	ESC-10	96.46%	-

TAT—Audio Tagging, mAP—mean average precision, AUC—area under the receiver operating characteristic curve, MCC—Matthews Correlation Coefficient, FDR—False Discovery rate, FM—Fowlkes-Mallows index.

**Table 8:** Summary of the urban sound datasets found in this literature review.

Reference	Name	Size	#Classes	Duration	Description
Salamon et al. [68]	UrbanSound8k	8732	10	≤4 s	Contains the metadata, Imbalanced classes
Piczak [69]	ESC-50	2000	30	≤5 s	Contains the metadata, Balanced classes
Piczak [69]	ESC-10	400	10	≤5 s	Contains the metadata, Balanced classes
Piczak [69]	ESC-US	250,000	-	≤5 s	Unlabeled Dataset
Koizumi et al. [70]	DCASE Task 2	8	-	≤10 s	Mechanical Anomalous Sounds
Cao et al. [71]	CREMA-D	7442	-	≤10 s	A selection of 12 sentences with emotions
Gemmeke et al. [72]	AudioSet	+2 Million	632	≤10 s	A large set of annotated sound categories
Mesaros et al. [73]	TUT Sound Event	24	15	≤15 s	Chopped into small samples
Rachman et al. [74]	MIREX	903	15	-	Mood Dataset for Emotion Classification
Fonseca et al. [75]	FSD50K	51,197	200	≤30 s	Human-labeled sound events

## Data Availability

Not applicable.

## Abbreviations

1D	one-dimensional
2D	two-dimensional
AAML	Additive Angular Margin Loss
ANN	Artificial Neural Network
APNet	Audio Prototype Network
AST	Audio Spectrogram Transformer
B-GRU	Bidirectional Gated Recurrent Unit
BERT	Bidirectional Encoder Representations from Transformers
BLSTM	Bidirectional Long-Short Term Memory
CENS	Chroma Energy Normalized Statistics
CNN	Convolutional Neural Network
CNN-Transformer	Convolutional Neural Network Transformer
CQT	Constant Q-transform
CRNN	Convolutional Recurrent Neural Networks
dB	decibel
DCNN	Deep Convolutional Neural Network
DeiT	Data efficiency image Transformer
DenseNet	Dense Convolutional Network
DL	Deep Learning
ERDF	European Regional Development Fund
ESC	Environmental Sound Classification
GFCC	Gammatone Frequency Cepstral Coefficient
GMM	Guassian Mixture Model
HMM	Hidden Markov Model
HPSS	Harmonic Percussive Source Separation
k-NN	k-Nearest Neighbor
LSTM	Long-Short Term Memory
M2M-AST	Many-to-Many Audio Spectrogram Transformer
MFCC	Mel Frequency Cepstral Coefficients
MIL-NCE	Multiple Instance Learning NCE
ML	Machine Learning
MSE	Mean Squared Error
NCE	Noise Contrastive Estimation
PRISMA	Preferred Reporting Items for Systematic Reviews and Meta-Analyses
RBF	Radial Basis Function
ResNet	Residual Neural Network
RGB	Red-Green-Blue
RNN	Recurrent Neural Network
SIF	Spectrogram Image Features
SSLS	Sound Source Localization and Separation
SSSC	Sound Source Separation and Classification
STFT	Short-Term Fourier Transformation
SVM	Support Vector Machine
TFCNN	Temporal-frequency attention based Convolutional Neural Network
VATT	Video-Audio-Text Transformer
ViT	Vision Transformer
VQ-VAE	Vector-quantized varitional autoencoders
YOHO	You Only Hear Once
